# Kidneys for Sale: Are We There Yet? (Commentary on Kidneys for Sale: Empirical Evidence From Iran)

**DOI:** 10.3389/ti.2022.10635

**Published:** 2022-06-24

**Authors:** Kyle R. Jackson, Christine E. Haugen, Dorry L. Segev

**Affiliations:** ^1^ Department of Surgery, Johns Hopkins University, Baltimore, MD, United States; ^2^ Department of Surgery, New York University, New York, NY, United States

**Keywords:** kindey, transplant, organ donation, compensation, Iran, debate

Direct financial compensation through organized markets has been proposed as one strategy to increase the number of potential organ donors. However, this controversial practice is legal in only one country in the world—Iran; thus, there are limited data to demonstrate how this type of system might practically function. In a new report, Moeindarbari and Feizi provide granular real-world data on the demographics of kidney vendors and recipients from 2011 to 2018 in the kidney market in Mashhad, Iran [1]. This study provides valuable insights from a direct financial compensation program for organ donation and helps contextualize the debate surrounding this controversial issue.

Most prior attempts to quantify the potential impact of direct financial compensation for donors have been limited to surveys or structured interviews about hypothetical compensation and willingness to donate, rather than the real-world data presented in this article [2–7]. These have generally found that direct financial compensation to donors would likely increase the number of people who would donate an organ. In one web-based survey of members of the Canadian general public, 54% of people who would not consider donation to a relative without any compensation would actually change to being willing to consider donation for a $10,000 payment [3]. Even among people who would already consider donation to a family member or a friend, a payment of $50,000 would make 60% of people even more likely to donate a kidney in a study from the United States [4].

The data presented by Moeindarbari and Feizi confirm that, even in a partially regulated organ sales market, donors are younger than recipients and have fewer years of education. This potentially validates previous concerns of donor exploitation and socio-economic inequalities that have been shown across many countries [8–10]. For example, the Phillipine Organ Donation Program allowed for direct financial payment to donors from 2002 to 2008, and 78% of donors did not have a single follow-up visit post-donation [10]. Importantly, Moeindarbari and Feizi point out that in addition to the market price set by the government for a kidney, the recipients are allowed to pay donors, which seems to undermine the idea of a “regulated” market and further engenders donor exploitation. The authors outlined policy recommendations and improvements moving forward to more fairly consider the market value of a kidney in Iran. However, we would suggest that we are not there yet: before we go down the road of commercial sales, there are many other methods to improve altruistic organ donation that have been underexplored and underutilized.

An increase in altruistic living and deceased donation could eliminate the need for commercial organ sales entirely. After the Israeli government criminalized organ brokering, altruistic living donation rose by 339% over 10 years [11]. Similar results were seen after the Pakistani government banned commercial transplants [12]. Iran has the opportunity to increase deceased donation efforts, given that deceased donors have only increased from 4% to 10% in 30 years [13]. Other ways to improve altruistic donation include the removal of disincentives to donate (such as expenses linked to donation, travel expenses, and lost wages) and removal of HLA, ABO, and other incompatibility barriers to living donation through paired exchange [14]. Other countries have shown these methods can substantially increase the access to living donation without commercial markets.

Even beyond simply removing disincentives to living donation, there are a number of other strategies that have been developed to increase the number of living donor kidney transplants being performed. For example, the Live Donor Champion program trains a friend, family member, or community member to advocate on behalf of a transplant candidate to identify a potential living donor and has been shown to increase the number of potential living donors who come forward to donate [15]. Other focused interventions have been developed to directly increase the number of donors who come forward for people who are racial/ethnic minorities or socioeconomically disadvantaged, although these have not been universally effective [16, 17]. In a randomized controlled trial of 145 African American kidney transplant candidates in the United States, 82% of candidates who received house calls (structured educational sessions delivered to candidates, family, and invited guests in their own home) received at least one donor inquiry compared to 47% of candidates who received traditional individual counseling in the transplant clinic [16]. In another study of 300 African American kidney transplant candidates in the United States, candidates were randomized to receive additional education from a transplant social worker (with or without living donor financial assistance) [TALKS] or to usual care [17]. The TALKS program was designed specifically to address issues precluding living donation raised by African Americans in prior studies. Although the TALKS intervention did not lead to an increase in living kidney donation, 99% of candidates who received the intervention reported a high degree of satisfaction with the intervention. Continued expansion and development of targeted interventions to increase living donation could help avoid the need for direct financial payment of living donors.

In summary, the data provided by Moeindarbari and Feizi shed both positive and negative light on how a regulated system of direct financial payment to organ donors actually functions. While such a system would likely increase the number of transplants performed, major concerns remain, and we would suggest that expanding systems designed to support and compensate donors for actual incurred expenses could substantially expand the number of donors without needing to directly provide payments for living donors. Additionally, continuing to innovate and expand the utilization of targeted interventions to increase living donation could also help avoid the need for direct financial compensation for living donors. Ultimately, we agree with the authors that careful study of this controversial topic is critical to ensuring protection of living donors.

## References

[1] Moeindarbara T Feizi M. Kidneys for Sale: Empirical Evidence from Iran. Transpl Int (2022). 10.3389/ti.2022.10178 PMC9266983 35812160

[2] Barnieh L Gill JS Klarenbach S Manns BJ. The Cost-Effectiveness of Using Payment to Increase Living Donor Kidneys for Transplantation. Clin J Am Soc Nephrol (2013) 8:2165–73. 10.2215/cjn.03350313 24158797 PMC3848395

[3] Barnieh L Klarenbach S Gill JS Caulfield T Manns B. Attitudes toward Strategies to Increase Organ Donation: Views of the General Public and Health Professionals. Clin J Am Soc Nephrol (2012) 7:1956–63. 10.2215/cjn.04100412 23024166 PMC3513747

[4] Peters TG Fisher JS Gish RG Howard RJ. Views of US Voters on Compensating Living Kidney Donors. JAMA Surg (2016) 151:710–6. 10.1001/jamasurg.2016.0065 27007405

[5] Elías JJ Lacetera N Macis M. Paying for Kidneys? A Randomized Survey and Choice Experiment. Am Econ Rev (2019) 109:2855–88. 10.3386/w25581

[6] Halpern SD Raz A Kohn R Rey M Asch DA Reese P. Regulated Payments for Living Kidney Donation: an Empirical Assessment of the Ethical Concerns. Ann Intern Med (2010) 152:358–65. 10.7326/0003-4819-152-6-201003160-00005 20231566 PMC2865248

[7] Held PJ McCormick F Ojo A Roberts JP. A Cost-Benefit Analysis of Government Compensation of Kidney Donors. Am J Transpl (2016) 16:877–85. 10.1111/ajt.13490 PMC5057320 26474298

[8] Gill J Dong J Rose C Johnston O Landsberg D Gill J. The Effect of Race and Income on Living Kidney Donation in the United States. J Am Soc Nephrol (2013) 24:1872–9. 10.1681/asn.2013010049 23990679 PMC3810084

[9] Zhang Y Gerdtham U-G Rydell H Jarl J. Socioeconomic Inequalities in the Kidney Transplantation Process: A Registry-Based Study in Sweden. Transplant Direct (2018) 4:e346. 10.1097/txd.0000000000000764 PMC5811275 29464207

[10] Padilla BS. Regulated Compensation for Kidney Donors in the Philippines. Curr Opin Organ Transpl (2009) 14:120–3. 10.1097/mot.0b013e328329256f 19469027

[11] Kurleto P Skorupska-Król A Broniatowska E Bramstedt KA. Exploring the Motives of Israeli Jews Who Were Living Kidney Donors to Strangers. Clin Transpl (2020) 34:e14034. 10.1111/ctr.14034 32652718

[12] Rizvi AHS Naqvi AS Zafar NM Ahmed E. Regulated Compensated Donation in Pakistan and Iran. Curr Opin Organ Transpl (2009) 14:124–8. 10.1097/mot.0b013e328326f6ef 19469028

[13] Malekshahi A MortezaNejad HF Taromsari MR Gheshlagh RG Delpasand K. An Evaluation of the Current Status of Kidney Transplant in Terms of the Type of Receipt Among Iranian Patients. Ren Replace Ther (2020) 6:66. 10.1186/s41100-020-00314-8

[14] Segev DL Gentry SE Warren DS Reeb B Montgomery RA. Kidney Paired Donation and Optimizing the Use of Live Donor Organs. JAMA (2005) 293:1883–90. 10.1001/jama.293.15.1883 15840863

[15] Garonzik-Wang JM Berger JC Ros RL Kucirka LM Deshpande NA Boyarsky BJ Live Donor champion: Finding Live Kidney Donors by Separating the Advocate from the Patient. Transplantation (2012) 93:1147–50. 10.1097/tp.0b013e31824e75a5 22461037 PMC3374007

[16] Rodrigue JR Paek MJ Egbuna O Waterman AD Schold JD Pavlakis M Making House Calls Increases Living Donor Inquiries and Evaluations for Blacks on the Kidney Transplant Waiting List. Transplantation (2014) 98:979–86. 10.1097/tp.0000000000000165 24825528 PMC4218873

[17] Boulware LE Sudan DL Strigo TS Ephraim PL Davenport CA Pendergast JF Transplant Social Worker and Donor Financial Assistance to Increase Living Donor Kidney Transplants Among African Americans: The TALKS Study, a Randomized Comparative Effectiveness Trial. Am J Transpl (2021) 21:2175–87. 10.1111/ajt.16403 PMC12533414 33210831

